# Supplementary material for: Safeguarding spermatogenesis from retrotransposon insertions by forming ecDNA
Source: bioRxiv. 2025 May 15:2025.05.11.653319. Preprint. [Version 1] doi: 10.1101/2025.05.11.653319 (PMC12132167; doi:10.1101/2025.05.11.653319)
Supplement: Supplement 1 [file NIHPP2025.05.11.653319v1-supplement-1.pdf]

bioRxiv preprint doi: https://doi.org/10.1101/2025.05.11.653319; this version posted May 15, 2025. The copyright holder for this preprint (which was not certified by peer review) is the author/funder, who has granted bioRxiv a license to display the preprint in perpetuity. It is made available under aCC-BY 4.0 International license.

Supplemental Figure 1

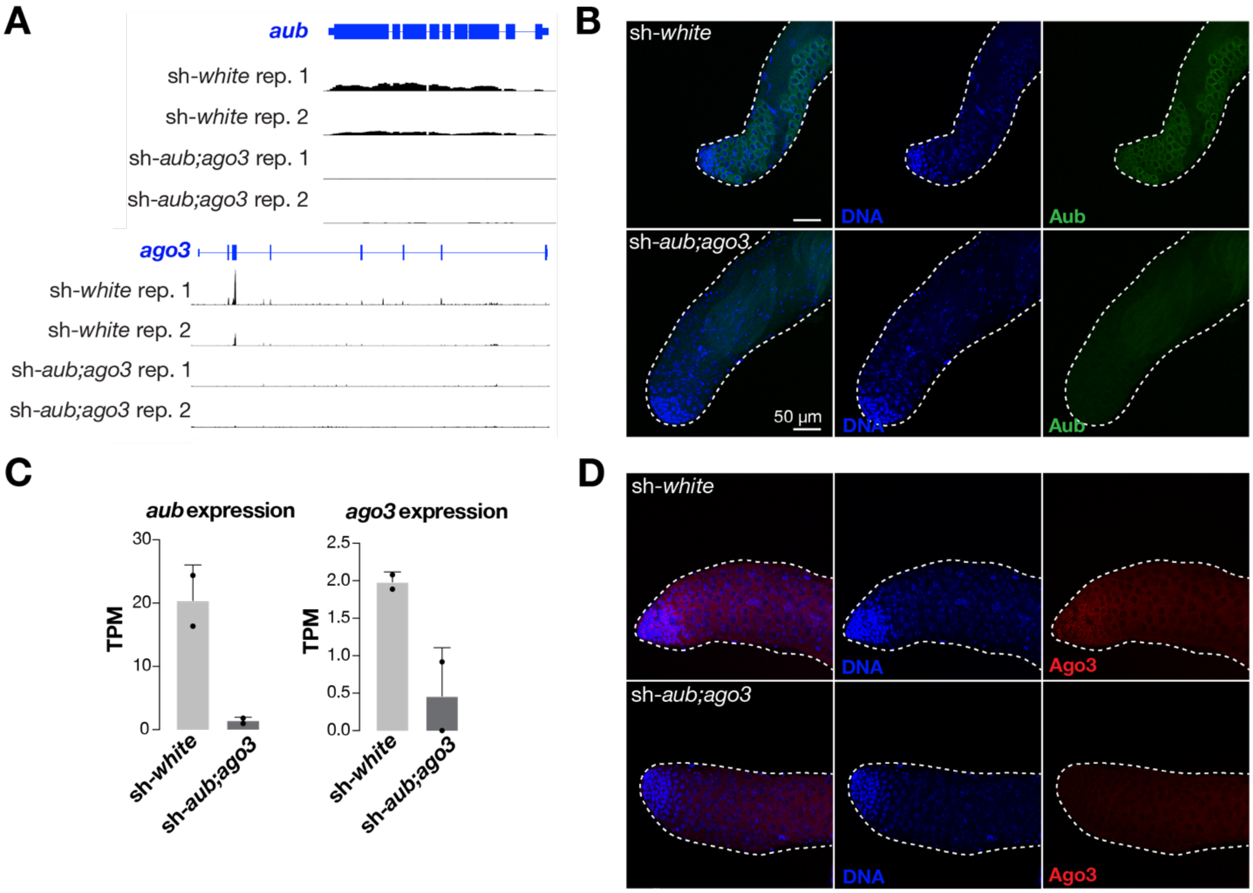

Supplemental Figure 1. Aub and Ago3 are efficiently depleted by RNAi in the testis. A. UCSC browser display of *aubergine* and *argonaute3* RNA expression from replicates of sh-*white* and sh-*aub;ago3* testes. **B.** Quantification of *aubergine* and *argonaute3* RNA expression from RNA sequencing. **C.** Immunofluorescence staining for Aub and Ago3 at the apical tip of testes dissected from 3-day-old flies. The sh-*aub* and sh-*ago3* constructs in these flies are driven by MTD-Gal4.

bioRxiv preprint doi: https://doi.org/10.1101/2025.05.11.653319; this version posted May 15, 2025. The copyright holder for this preprint (which was not certified by peer review) is the author/funder, who has granted bioRxiv a license to display the preprint in perpetuity. It is made available under aCC-BY 4.0 International license.

Supplemental Figure 2

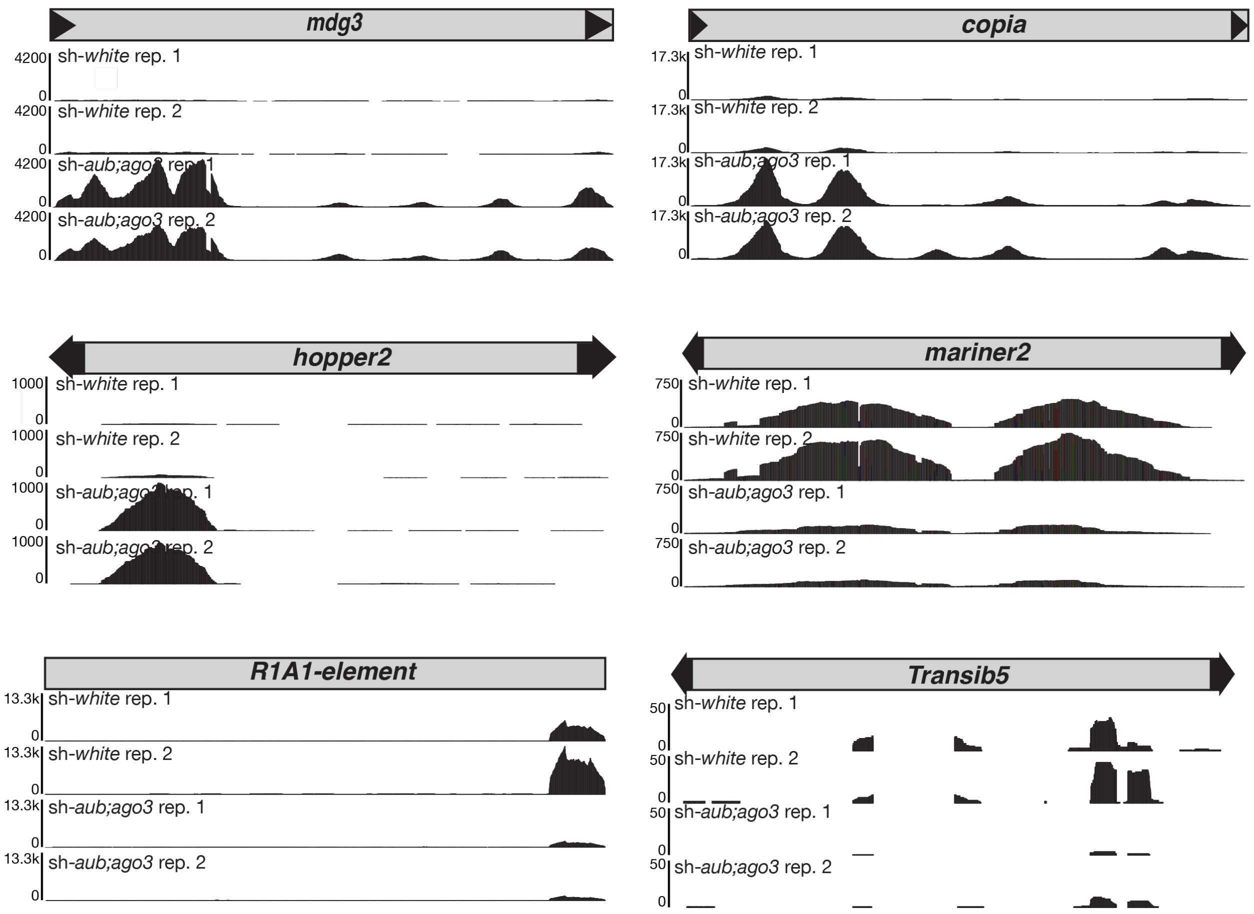

Supplemental Figure 2. Visualization of RNA-seq reads for transposons that are differentially expressed in control and transposon derepressed testes. RNA-seq reads from across the length of each transposon are visualized using IGV.

bioRxiv preprint doi: https://doi.org/10.1101/2025.05.11.653319; this version posted May 15, 2025. The copyright holder for this preprint (which was not certified by peer review) is the author/funder, who has granted bioRxiv a license to display the preprint in perpetuity. It is made available under aCC-BY 4.0 International license.

Supplemental Figure 3

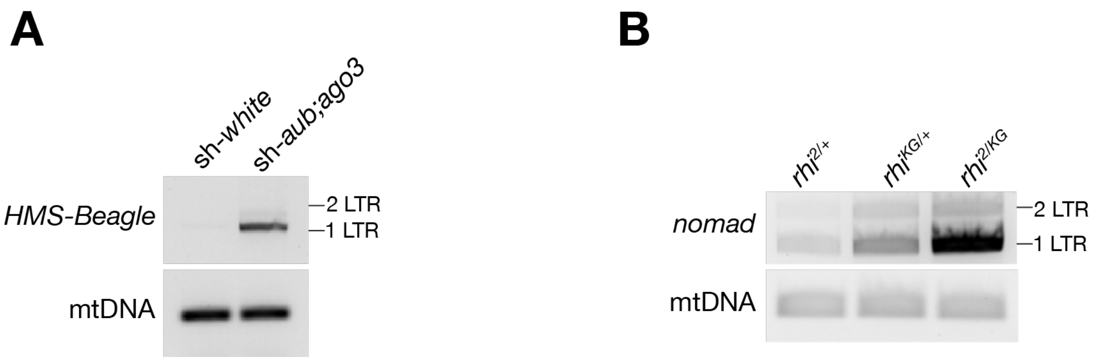

Supplemental Figure 3. Transposon ecDNA can be detected by divergent PCR. A. Divergent PCR for *HMS-Beagle* from control and transposon derepressed testes. Input for PCR was DNA from 3-day-old testes with linear DNA digested. **B.** Divergent PCR for *nomad* ecDNA from 1-day-old testes of heterozygous control testes or *rhino* mutant testes. Linear DNA was digested from the sample.

bioRxiv preprint doi: https://doi.org/10.1101/2025.05.11.653319; this version posted May 15, 2025. The copyright holder for this preprint (which was not certified by peer review) is the author/funder, who has granted bioRxiv a license to display the preprint in perpetuity. It is made available under aCC-BY 4.0 International license.

Supplemental Figure 4

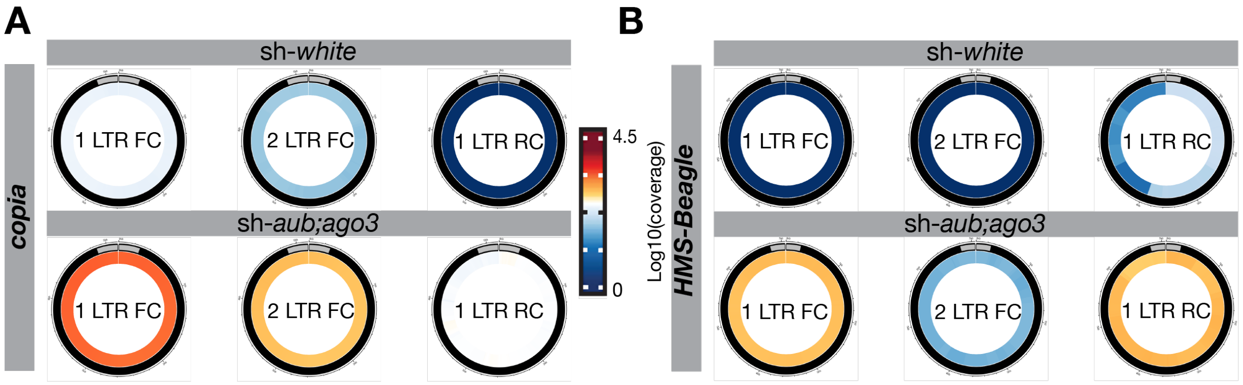

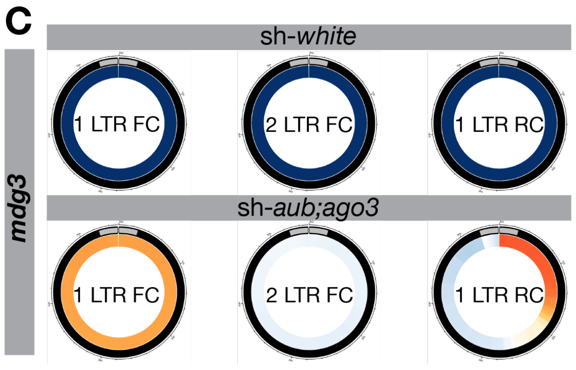

D

	1 LTR FC	2 LTR FC	1 LTR rearranged	non LTR rearranged
nomad	43.9%	1.4%	52.5%	2.3%
copia	73.9%	12.1%	11.3%	2.7%
HMS-Beagle	30.2%	0.3%	63.0%	6.5%
mdg3	19.0%	3.8%	76.4%	0.8%

Supplemental Figure 4. LTR retrotransposons predominantly make 1-LTR ecDNA. A-C. Circos plots of different types of LTR retrotransposon ecDNA and their abundance in testes from 3-day-old flies. Plots are from one replicate of ecDNA sequencing, each with a similar amount of mitochondrial DNA. Gray boxes signify the LTR region of the retrotransposon. FC = full-length circle. RC = rearranged circle. **D.** Chart reporting the percentage of ecDNA circles that correspond to each type of circle from sh-*aub;ago3* testes ecDNA sequencing. This is average data from three replicates of sh-*aub;ago3* ecDNA sequencing. Each replicate was normalized by the mitochondrial DNA content from that sample.

bioRxiv preprint doi: https://doi.org/10.1101/2025.05.11.653319; this version posted May 15, 2025. The copyright holder for this preprint (which was not certified by peer review) is the author/funder, who has granted bioRxiv a license to display the preprint in perpetuity. It is made available under aCC-BY 4.0 International license.

Supplemental Figure 5

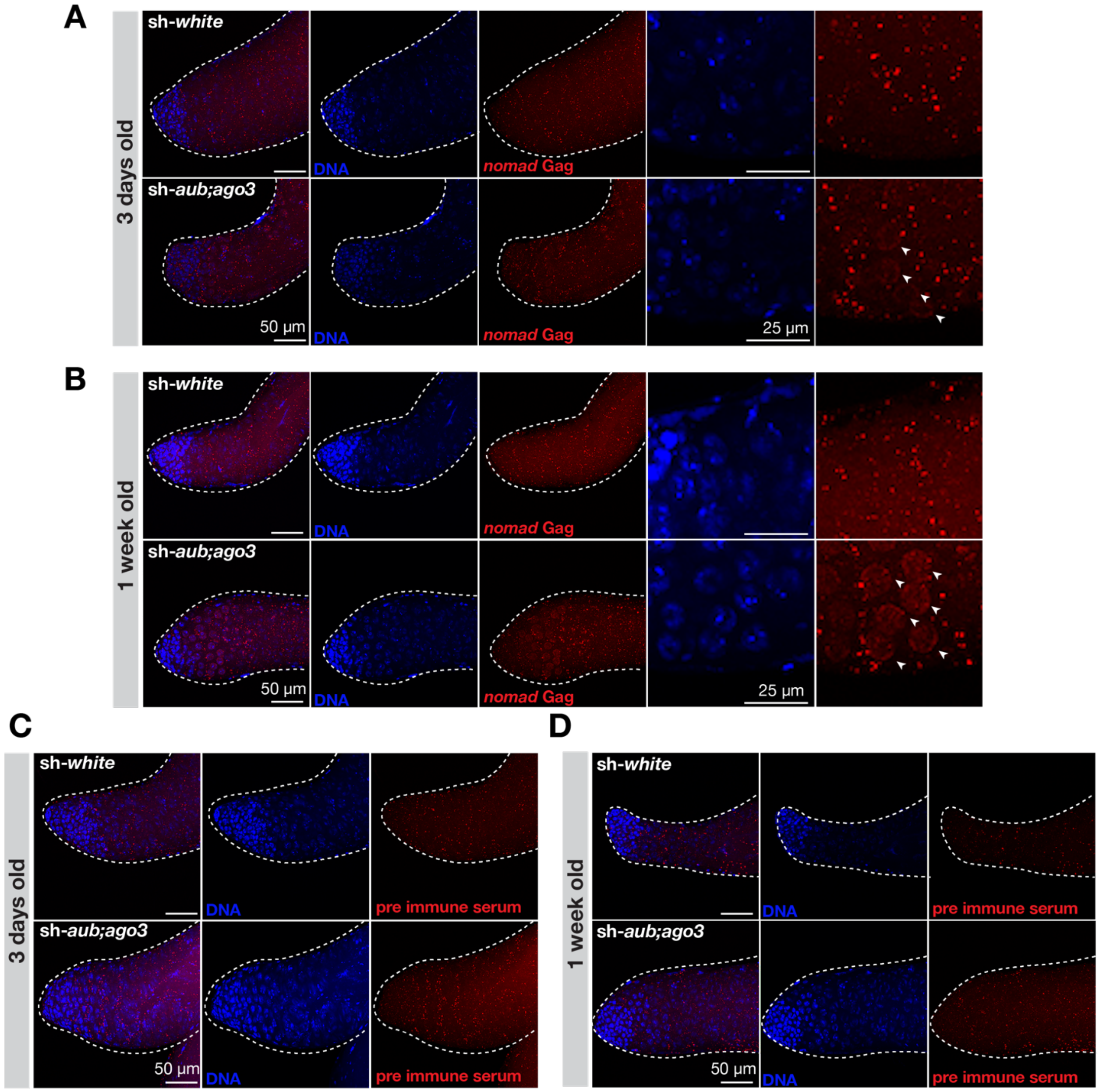

Supplemental Figure 5. *nomad* Gag is produced in spermatocytes. A. *nomad* Gag immunostaining at the apical tip of testes dissected from 3-day-old flies. **B.** *nomad* Gag immunostaining at the apical tip of testes dissected from 1-week-old flies. **C.** Three-day-old testes incubated with pre immune serum in replacement of *nomad* Gag antibody. **D.** Testes from 1-week-old flies incubated with pre immune serum in replacement of *nomad* Gag antibody.

bioRxiv preprint doi: https://doi.org/10.1101/2025.05.11.653319; this version posted May 15, 2025. The copyright holder for this preprint (which was not certified by peer review) is the author/funder, who has granted bioRxiv a license to display the preprint in perpetuity. It is made available under aCC-BY 4.0 International license.

Supplemental Figure 6

A

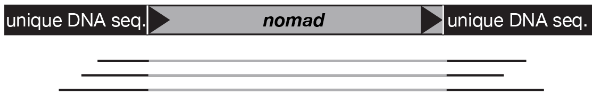

B

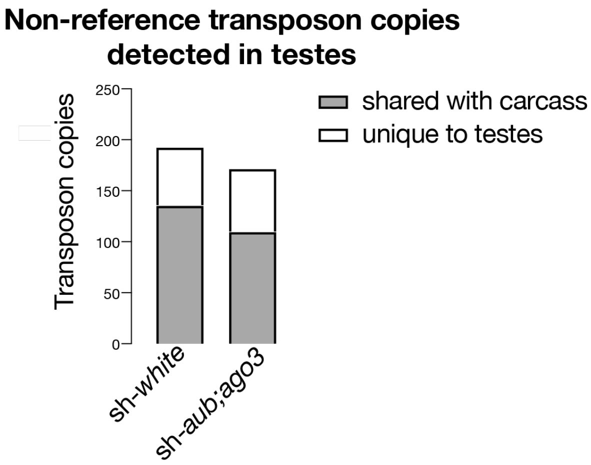

C

non-reference transposon copies

	nomad	*mdg3*	*copia*	*HMS-Beagle*
sh-*white* testes	0	2	4	2
sh-*aub;ago3* testes	1	2	3	1

Supplemental Figure 6. LTR retrotransposons that make ecDNA in the testes rarely integrate. A. Model of the types of reads that were used for panels B and C. **B.** Total transposon copies detected by the TLDR pipeline in the testes of control and transposon derepressed flies. This pipeline requires three or more reads to support each copy with flanking sequence. **C.** Transposon copies detected in sh-*white* and sh-*aub;ago3* testes for LTR retrotransposons that make significantly more ecDNA upon derepressing transposons.

bioRxiv preprint doi: https://doi.org/10.1101/2025.05.11.653319; this version posted May 15, 2025. The copyright holder for this preprint (which was not certified by peer review) is the author/funder, who has granted bioRxiv a license to display the preprint in perpetuity. It is made available under aCC-BY 4.0 International license.

Supplemental Figure 7

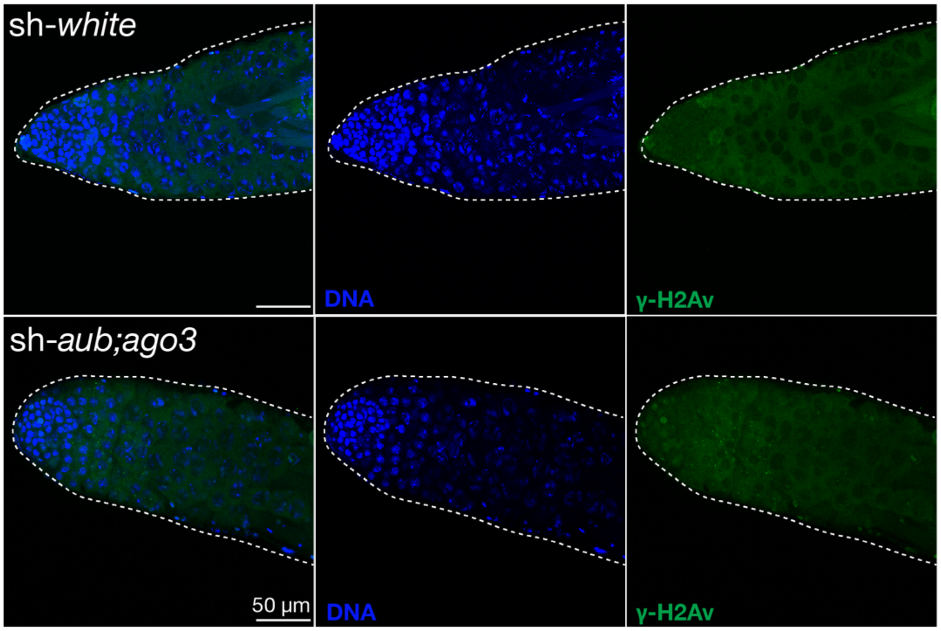

Supplemental Figure 7. There is minimal DNA damage in transposon derepressed testes. γ-H2Av immunostaining for DNA double-strand breaks at the apical tip of testes dissected from 3-day-old flies.

bioRxiv preprint doi: https://doi.org/10.1101/2025.05.11.653319; this version posted May 15, 2025. The copyright holder for this preprint (which was not certified by peer review) is the author/funder, who has granted bioRxiv a license to display the preprint in perpetuity. It is made available under aCC-BY 4.0 International license.

Supplemental Figure 8

A

Total non-reference transposon copies

	**Total transposons**
sh-*white* larvae	841
sh-*aub;ago3* larvae	567

B

Non-reference transposon copies

	mdg3	***copia***	***HMS-Beagle***
sh-*white* larvae	16	34	12
sh-*aub;ago3* larvae	12	18	7

Supplemental Figure 8. Offspring from males with transposons derepressed in the germline do not have an increase in transposon genomic transposon copies. A. Total non-reference transposon copies detected in the offspring from crosses of wild-type females crossed with either a control male (MTD-Gal4>sh-*white*) or male with transposons derepressed (MTD-Gal4>sh-*aub;ago3*) in the germline. Transposons have at least 3 reads supporting each copy and flanking sequence. **B.** Non-reference transposon copies from LTR retrotransposons that make ecDNA.

bioRxiv preprint doi: https://doi.org/10.1101/2025.05.11.653319; this version posted May 15, 2025. The copyright holder for this preprint (which was not certified by peer review) is the author/funder, who has granted bioRxiv a license to display the preprint in perpetuity. It is made available under aCC-BY 4.0 International license.

Supplemental Material Description

Supplemental Figure S1- This figure shows that two primary components of the piRNA pathway, Aub and Ago3, are able to be depleted by RNAi. This is foundational for the paper because this is the model of transposon de-repression used throughout the paper.

Supplemental Figure S2- This figure shows a more detailed view of how disrupting the piRNA pathway can change transposon RNA expression.

Supplemental Figure S3- This figure shows that transposon ecDNA can be detected by PCR in two different genetic models of transposon activation (depleting Aub and Ago3 by RNAi/ mutating *rhino*).

Supplemental Figure S4- This figure supplements a main conclusion that LTR retrotransposon ecDNA can integrate into itself (shown by 1-LTR rearranged circle coverage plots). It also shows the proportion of each circle type that is made by other transposons in addition to *nomad*.

Supplemental Figure S5- This figure supports the conclusion that *nomad* products ae produced in the spermatocytes by showing that *nomad* Gag protein is produced in those cells upon Aub/Ago3 depletion.

Supplemental Figure S6- This figure supports the conclusion that transposons do not abundantly integrate into the genomes of testes cells upon Aub/Ago3 depletion.

Supplemental Figure S7- This figure supports the conclusion that transposition is rare in the testes by showing minimal DNA double-strand breaks in the testes of both sh-*white* and sh-*aub;ago3* testes.

Supplemental Figure S8- This figure supports the conclusion that transposition is rare in the testes even when transposons are derepresses, as there is not an increase in transposon content in the offspring of sh-*aub;ago3* males.

Supplemental Table S1- This table reports the primers used for divergent PCR for Fig. 3 and Supplemental Fig. 3.

Supplemental Table S2- This table reports the RNA FISH probe sets for *nomad* and *copia* used in Fig. 4.

Supplemental Table S3- This table provides basic information (library preparation kit, read statistics, etc.) about the sequencing data used in this manuscript.

bioRxiv preprint doi: https://doi.org/10.1101/2025.05.11.653319; this version posted May 15, 2025. The copyright holder for this preprint (which was not certified by peer review) is the author/funder, who has granted bioRxiv a license to display the preprint in perpetuity. It is made available under aCC-BY 4.0 International license.

Primer	Sequence	PCR product size	Figure
nomad-ecDNA-1-F	CACATAACGGCCAGCAACTT	1721 bp (2 LTRs), 1203 bp (1 LTR)	Fig. 3C
nomad-ecDNA-1-R	GCAGTGGGTTGATCGTGTTT		Fig. 3C
mtDNA-F	ATTGGCTGGAATACCTCGAC	207 bp	Fig. 3C, Supp. Fig. 3A,B
mtDNA-R	GCTGGTGGAGTATTTTGGTA		Fig. 3C, Supp. Fig. 3A,B
HMS-Beagle-ecDNA-F	ACAGAAACTGTGATCCACCA	1797 bp (2 LTRs), 1531 bp (1 LTR)	Supp. Fig. 3A
HMS-Beagle-ecDNA-F	TACCGTAGCTCCAGAAAAAT		Supp. Fig. 3A
nomad-ecDNA-2-F	AAACTGGAGGAGGCTAGCTG	1962 bp (2 LTRs), 1444 bp (1 LTR)	Supp. Fig. 3B
nomad-ecDNA-2-R	GCAGTGGGTTGATCGTGTTT		Supp. Fig. 3B

bioRxiv preprint doi: https://doi.org/10.1101/2025.05.11.653319; this version posted May 15, 2025. The copyright holder for this preprint (which was not certified by peer review) is the author/funder, who has granted bioRxiv a license to display the preprint in perpetuity. It is made available under aCC-BY 4.0 International license.

**Target**	***nomad***	***copia***
**Dye**	**Quasar 570**	**Quasar 570**
**Orientation**	**5' to 3'**	**5' to 3'**
1	tcctgacgaagatcgcaaga	atttcaactgcaacaccagc
2	tgacaaccaacatgctgtgg	tcggctaaaagagccctaat
3	gcgggtcgttgttttattaa	tactttttgcacaacgctct
4	gtatcgtacaacttttgcgc	gtcgcttaggtactctatta
5	atcttttgcactcgcgaatt	ttctcaagaatctgacgcgc
6	cattgagggactcgctaaga	cgccaaactttttcgttcat
7	gcaagacaattgccctatac	atctcactcgatagcttcag
8	aagcgtacgtaagtctcgtt	tccatctcttctatttttgc
9	ttgactacttcacgagggtg	tttcttgcttgtatcgttgt
10	aaagccttgtctctataggt	acagtggtgacacttgactt
11	gtgtttggaacaatctctcc	cttttttaatgtggccttct
12	attacattctgcaatcccat	aaacgcaatgccgtgtgatg
13	gattggtaagattgacccga	gaatcaaggacaaacccgca
14	caaggttatgtggagcatct	ccactgcaatcttaagtgga
15	tctccgtagcatactctaag	cattccgtagtcggacaata
16	gaatttaccaactaggcggt	aattcgatcgacattcctgc
17	aggtaatatccgagtcgttc	cgaaatggttacaccgcttt
18	taagggctattagagggtcg	cctctcatgccataaacgaa
19	tttaggtttcttcgggacta	cttgcctgtttaccatttaa
20	tatctgggatggggtaagtg	atctcatatggggtctttga
21	gacggaggaactcgtacttt	agtatggcttcttattgtgc
22	ggtgcattcttcaaaccgaa	gttgcaccaaacactctcaa
23	ccagtgtgtgtcataatctt	gggttcatagcccacaaaaa
24	acttctcaaggttcacttgg	tttcctactgtcattcggaa
25	ttaacagaggtcggaggagg	tttcctactgtcattcggaa
26	gaggtcatgcctagaaatct	catctcgctttcttttctta
27	aacatgggaacgccagtatt	tttaagtgctctgctgtttc
28	agcatttccttttcgatagt	gcctttttgtaattgtccaa
29	gcagttgtattcctctatac	gtgcaaccaatctagctttg
30	gctcaactggttaagctgag	gctacaggagcaaatgtctc
31	cctcgggattagcatctaaa	gtccgtaaattgccttattc
32	ggtgctcgcataagtatttt	acttcaaaccagcatctagc
33	ttcgctaagcagatggactg	ttcttgtcatatctcctgta

bioRxiv preprint doi: https://doi.org/10.1101/2025.05.11.653319; this version posted May 15, 2025. The copyright holder for this preprint (which was not certified by peer review) is the author/funder, who has granted bioRxiv a license to display the preprint in perpetuity. It is made available under aCC-BY 4.0 International license.

34	tactggacattcgcgggaaa	attttatcttcctgcatctc
35	tgaggaagtttgcagacgga	gtattgcagtcttcatctga
36	ggtagttaggaattggcgta	aatctgggcgtgtacaaagc
37	atgcttttgactgcagatgg	actctttttaagttctgcca
38	cgtagagtggaatctctcga	tatcgatagtgcccttcaaa
39	tttgaaggtagggagctcat	ataccctgttgtactttttc
40	tcgttaccgagtgaacggaa	ctactgagttctgtctcttt
41	ctcaattaagcccttgatgt	ctctcacggcttcaaatagg
42	atctggtcgtctaagttgtt	gctaatacagccttgattgt
43	actcgttggttacttcagta	ctcgtttatgacatgagggg
44	tgactgaccgagtaactgga	attctgaacttgctctctgg
45	aagagaggctagtagctcac	cgtaactccacaaatctcgc
46	tctaagtttgcggcaatctc	cttgcagcaaacccaatttg
47	tcctgacgaagatcgcaaga	gtgttaactgatccagcatt
48	tgacaaccaacatgctgtgg	atttcaactgcaacaccagc

bioRxiv preprint doi: https://doi.org/10.1101/2025.05.11.653319; this version posted May 15, 2025. The copyright holder for this preprint (which was not certified by peer review) is the author/funder, who has granted bioRxiv a license to display the preprint in perpetuity. It is made available under aCC-BY 4.0 International license.

RNA Sequencing					
Sample	Barcode sequence	Number of reads	Total bases (Mbases)	Mean quality score	%Bases >= 30
mtdGal4>sh-*white*	TTAGGC	38,837,960	11,651	35.17	90.03
mtdGal4>sh-*white*	TGACCA	114,176,869	34,253	35.6	91.96
mtdGal4>sh-*aub;ago3*	ACTTGA	54,148,304	16,244	35.63	92.16
mtdGal4>sh-*aub;ago3*	CAGATC	63,803,380	19,141	35.68	92.39

ecDNA Sequencing							
Sample	Barcode	Sequencing Kit	Number of reads	Read length N50	Median read length	Mean read length	Total bases
sh-*white* ecDNA rep. 1	barcode16	SQK-LSK114	106128	13475	2168	5505	584269829
sh-*white* ecDNA rep. 2	barcode17	SQK-LSK114	93700	15458	4915	8032	752688230
sh-*white* ecDNA rep. 3	barcode18	SQK-LSK114	101502	15021	5417	8173	829610849
sh-*aub;ago3* ecDNA rep. 1	barcode19	SQK-LSK114	157943	11751	1545	4432	700106416
sh-*aub;ago3* ecDNA rep. 2	barcode20	SQK-LSK114	180730	13496	1492	4777	863412071
sh-*aub;ago3* ecDNA rep. 3	barcode21	SQK-LSK114	220033	12570	2036	5333	1173626493

Genomic DNA Sequencing							
Sample	Barcode	Sequencing Kit	Number of reads	Read length N50	Median read length	Mean read length	Total bases
sh-*white* carcass	barcode02	SQK-RBK004	416487	6700	2786	4034	1680488921
sh-*aub;ago3* carcass	barcode04	SQK-RBK004	243014	12295	4555	7005	1702390396
sh-*white* testes	barcode05	SQK-RPB114	1914773	5393	4776	4879	9343693083
sh-*aub;ago3* testes	barcode06	SQK-RPB114	1842002	4772	4038	4227	7786804192
Offspring of sh-*white* males	barcode09	SQK-RBK004	454643	10529	3682	5869	2668694551
Offspring of sh-*aub;ago3* males	barcode12	SQK-RBK004	467828	7382	2812	4350	2035184544
